# Supplementary material for: Synthesis and Characterization of the Novel Nε-9-Fluorenylmethoxycarbonyl-l-Lysine N-Carboxy Anhydride. Synthesis of Well-Defined Linear and Branched Polypeptides
Source: Polymers (Basel). 2020 Nov 27;12(12):2819. doi: 10.3390/polym12122819 (PMC7759796; doi:10.3390/polym12122819)
Supplement: Supplementary file 1 [file polymers-12-02819-s001.pdf]

polymers

Supporting Information for Synthesis and Characterization of the Novel N^{ε}-9-Fluorenylmethoxycarbonyl-L-Lysine *N*-Carboxy Anhydride. Synthesis of Well-Defined Linear and Branched Polypeptidic Polymers Through their Orthogonal Deprotection

Varvara Athanasiou [1], Pandora Thimi [1], Melina Liakopoulou [1], Foteini Arfara [1], Dimitra Stavroulaki [1], Iro Kyroglou [1], Dimitrios Skourtis [1], Ioanna Stavropoulou [1], Panagiotis Christakopoulos [1], Maria Kasimatis [1], Panagiota G. Fragouli [2] and Hermis Iatrou [1,*]

[1] University of Athens, Department of Chemistry, Industrial Chemistry Laboratory, Panepistimiopolis, Zografou, Athens GR–15771, Greece;
[2] University of West Attica, DIDPE, Dyeing, Finishing, Dyestuffs and Advanced Polymers Laboratory, 250 Thevon Street, Athens GR–12241, Greece;
* Correspondence: iatrou@chem.uoa.gr; Tel.: +30-210-727-4330

Synthesis of N^{ε}-tert-butyloxycarbonyl-L-Lysine N-Carboxy Anhydride (N^{ε}-Boc-L-Lys NCA).

N^{ε}-Boc-L-Lys NCA was synthesized according to previously reported method [1,2]. Briefly, 17g of N^{α},N^{ε}-di-(*tert*-butyloxycarbonyl)-L-Lysine-OH*DCHA was dissolved in 150ml of ethyl acetate and a solution of H_2SO_4 1M was added dropwise. The solution became clear as an indication of the dicyclohexylamine salt removal from the amino acid. Then, 50ml milliq water was added to the solution and the organic phase was washed twice with ethyl acetate. The combined organic phases were dried with $MgSO_4$ and the free amino acid N^{α},N^{ε}-di-(tert-butyloxycarbonyl)-L-Lysine was obtained after filtration. The solid was dried on a high vacuum line overnight in a two-neck round bottom flask. Then 250ml dry ethyl acetate was distilled to the flask, placed in the fume hood and under argon atmosphere a solution of triphosgene in dry ethyl acetate was added dropwise for 30 minutes. The mixture was left to react for 10 minutes. Triethylamine diluted in dry ethyl acetate was subsequently added dropwise, and the solution was immersed in an ice-water bath for 2.5 hours. The precipitate was filtered, in order to remove the HCl salt of triethylamine, and the organic filtrate was washed with 6% NaCl solution until pH=4 of the aqueous phase was achieved. The organic phase was additionally washed twice with 0.5% $NaHCO_3$ (aqueous) until neutral pH. The organic phase was separated, dried over $MgSO_4$, filtered, and concentrated to approximately $^1/_3$ of the initial volume. The addition of 700ml dry hexane resulted in the precipitation of the desired product, which was filtered, dried, and recrystallized one more time with EtOAc/hexane. Finally, N^{ε}-Boc-L-Lys NCA was dried under high vacuum overnight and transferred into a glove box. The purity of N^{ε}-Boc-L-Lys NCA was confirmed by ^{1}H-NMR and FT-IR spectroscopy. Typical yield was 40.3%.

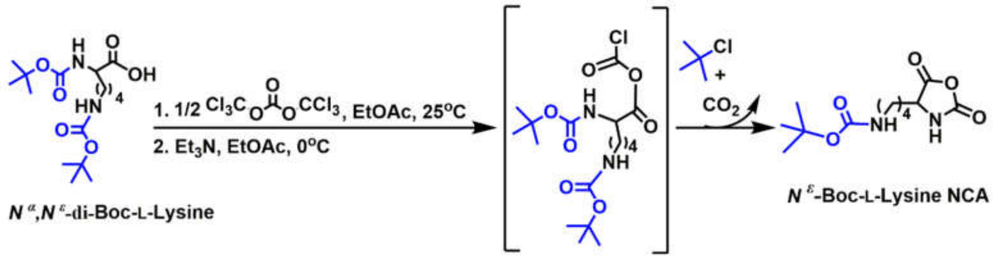

Scheme S1. Reactions used in synthesis of N^{ε}-Boc-L-Lys NCA.

Synthesis of N^{im}-trityl-L-Histidine N-Carboxy Anhydride (N^{im}-Trt-His-NCA).

According to a previous report of our group the synthesis of *N^{im}-Trt-His-NCA* was performed in two steps [3]. The first synthetic step involved the synthesis of the HCl salt of N^{im}-Trt-His-NCA, followed by removal of the HCl to obtain the pure monomer.

Synthesis of N^{im}-Trt-His-NCA·HCl. In a 500 mL round-bottom flask 10 g (20.1 mmol) of Boc-His(Trt)-OH were added and dried overnight under high vacuum. THF (150 mL) was distilled in the flask, giving a clear yellowish solution. The reaction flask was placed in an ice-bath, filled with argon and 1.7 mL (23.44 mmol) of thionyl chloride diluted in 50 mL of THF were added dropwise over a period of 20 minutes. By the end of addition of thionyl chloride, the solution became yellowish and a small aliquot was analyzed with IR. After 2.5 hours the solution was poured into 2 L of cold $(Et)_2O$ with precipitation of N^{im}-Trt-His-NCA·HCl as the major product. Finally, the solid was filtered (glass sintered filter 3) and then transferred to a 500 mL round bottom-flask and dried in HV. Recrystallization was conducted to the solid mixture, containing the HCl salt, free anhydride and the initial substrate by distilling 250 mL of ethyl acetate under HV. The flask containing the suspension was removed from HV and placed into a water bath at 45 °C for 1 hour, resulting in dissolution. The solution was then cooled to 0 °C with an ice bath and Trt-His NCA·HCl was formed as a precipitate, which was isolated as the only product after filtration. The NCA salt was transferred to another flask and dried overnight under HV (6.22 g = 13.52 mmol).

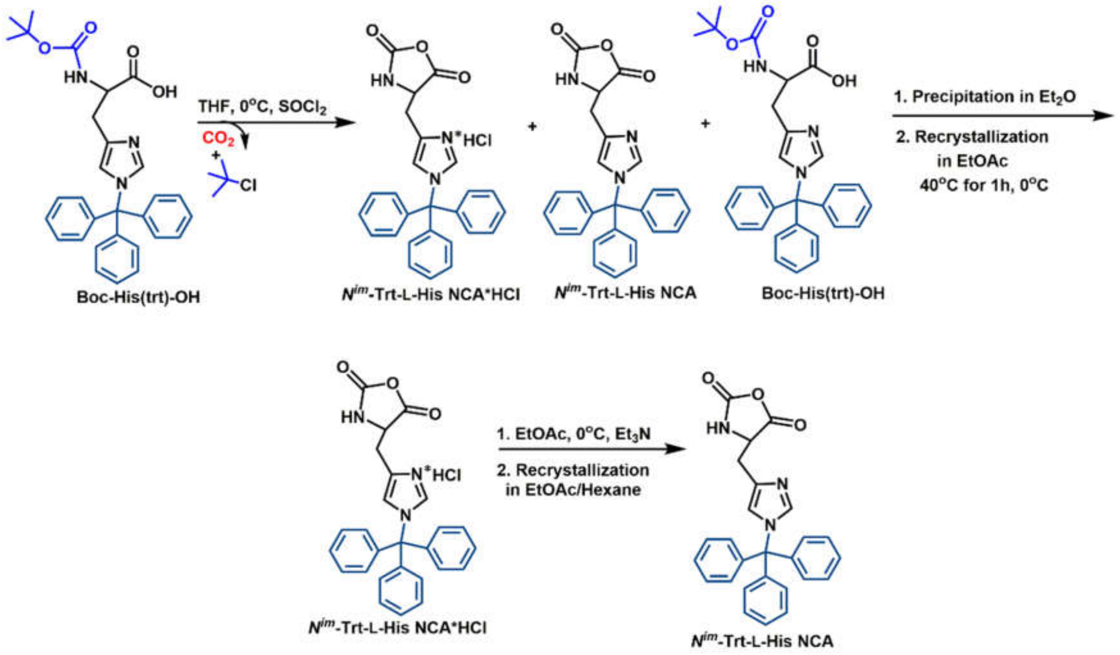

Scheme S2. Reactions used in synthesis of *N*^{im}-Trt-HIS-NCA.

***N*^{im}-Trt-His-NCA**. Subsequently, 200 mL of EtOAc were distilled into the flask, the flask was removed from HV, filled with argon and placed in an ice bath. At 0 °C, 1.88 mL (13.52 mmol) of stoichiometric amount of triethylamine dissolved in 50 mL of the same solvent was slowly added dropwise under vigorous stirring (duration of addition 1 hour). The resulting triethylamine hydrochloride was filtered off and the filtrate was poured into 1.5 L of non-solvent hexane in order to recrystallize the Trt-His-NCA. A second recrystallization occurred with a mixture of EtOAc–hexane (1:5) and the white solid precipitate Trt-His-NCA was isolated by filtration. Finally, *N*^{im}-Trt-His-NCA was dried under HV overnight and transferred into a glove box to afford 4.9 g. (11.57 mmol, 58% yield). The purity of *N*^{im}-Trt-His-NCA was confirmed by ^{1}H-NMR and FT-IR spectroscopy.

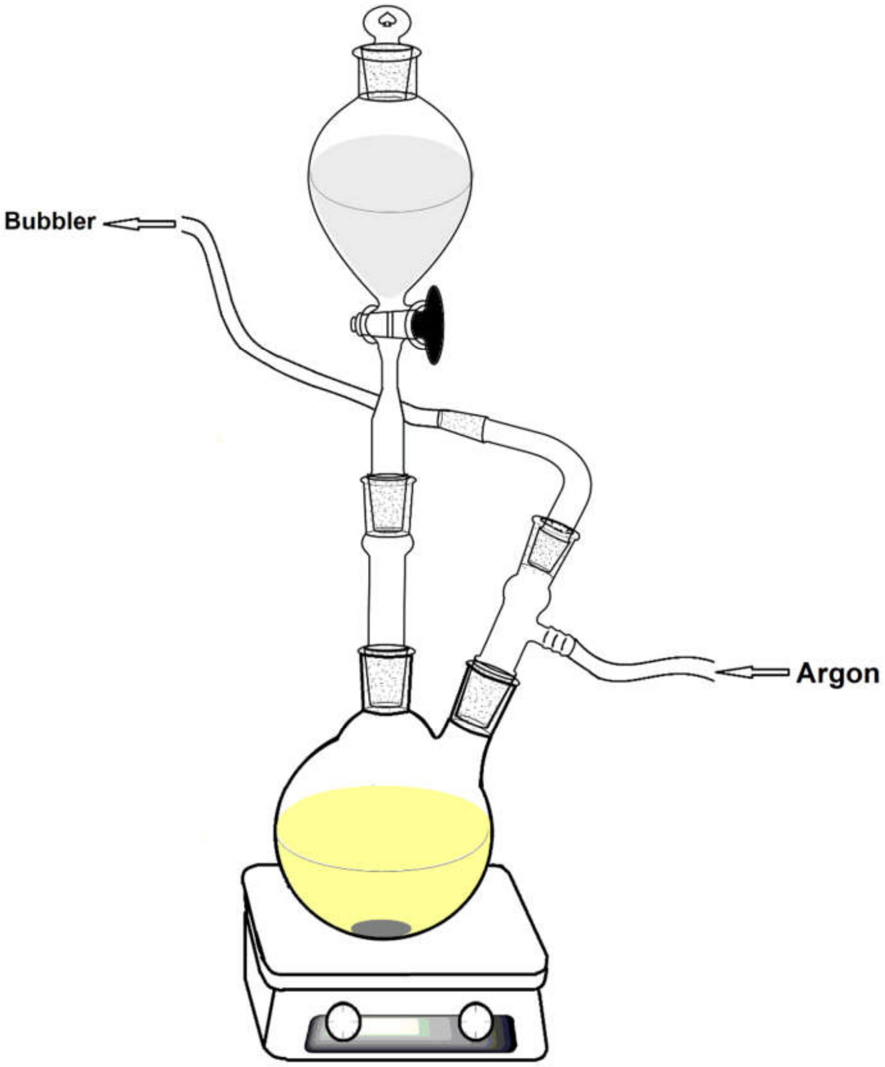

Scheme S3. A suitable pad used for the synthesis of *N*$^{\varepsilon}$-9-Fluorenylmethoxycarbonyl-L-Lysine (*N*$^{\varepsilon}$-Fmoc-L-Lys NCA) as well as of *N*^{im}-trityl-L-Histidine (*N*^{im}-Trt-HIS-NCA) N-Carboxy Anhydrides.

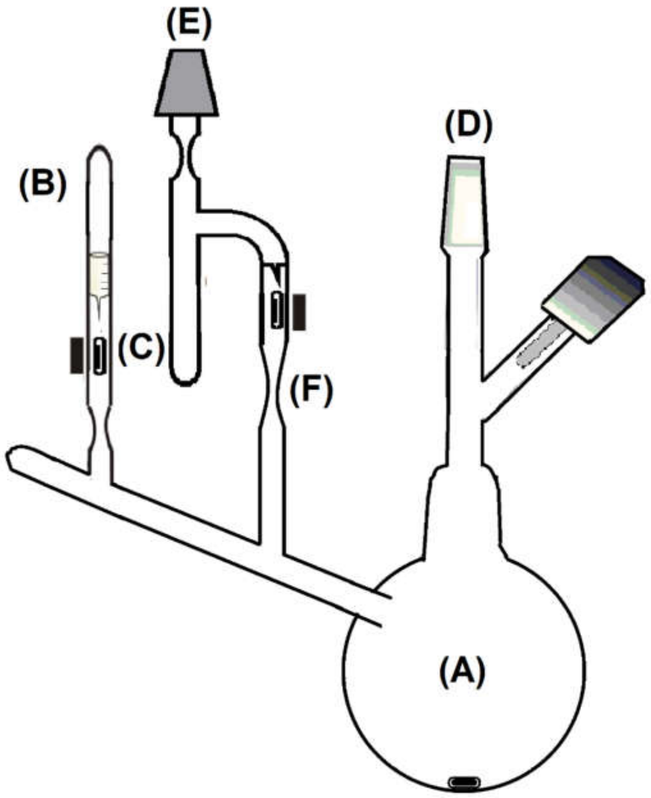

Scheme S4. The custom-made apparatus used for the synthesis of Poly(N^{ε}-Fmoc-L-Lys) homopolypeptide.

Deprotection mechanism of Fmoc protective groups with piperidine

Protective moieties based on 9-Fluorenylmethyl group are the most used protectors deprotected by β-elimination. 9-fluorenylmethyl protective groups started to be used in the 1970 and they are stable under acidic conditions but unstable to basic conditions and secondary amines. The aromatic rings activate the C-9 hydrogen atom and piperidine deprotonates the protecting moiety and further bind with it at the terminal carbon atom before the formation of the double bond. The addition product is insoluble in aqueous acid and cannot be removed by extraction. There is no gas production during the deprotection process indicating the binding between carbon dioxide and piperidine that form a salt. The 9-fluorenylmethoxycarbonyl protector is stable to acidolysis, so it is orthogonal to both benzyl and *tert*-butyl-based groups. Nevertheless, it is not orthogonal to benzyl-based protectors if the process of deprotection is reduction [4].

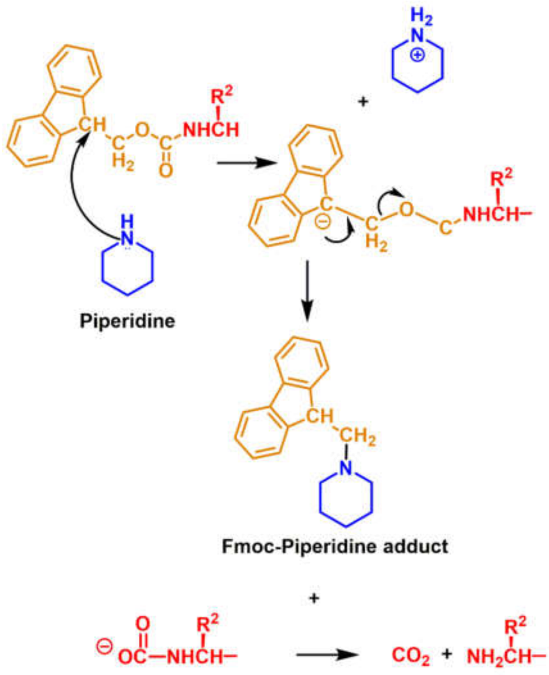

Scheme S5. Removal of the 9-fluorenylmethoxycarbonyl group by beta-elimination. Deprotonation is achieved by piperidine that subsequently forms a byproduct with the liberated moiety.

Characterization of N^ε^-Boc-L-Lys NCA

FT-IR measurement of the Boc-Lys(Boc)-OH was firstly took place (Figure S1A). A characteristic peak at 1645 cm^{-1} is observed, which is due to the vibration of carbonyl (C=O) of the amino acid of lysine. The wide peak from 3300–3800 cm^{-1} corresponds to the vibration of the primary amine in combination with the hydroxyl of the carboxyl group. The addition of triphosgene was followed by addition of triethylamine for the capture of hydrochloride produced during the reaction. Completion of the reaction was confirmed by FT-IR spectroscopy after 2.5 hours. (Figure S1B). The disappearance of the peak at 1645 cm^{-1} and the appearance of two new peaks in the carbonyl region at 1790 cm^{-1} and 1833 cm^{-1} indicate success in the synthesis of anhydride. Furthermore, the absence of the peak at 1650 cm^{-1} at the final spectrum (Figure S1C), which would be due to the vibration of the peptide bond, also confirm the success in NCA synthesis.

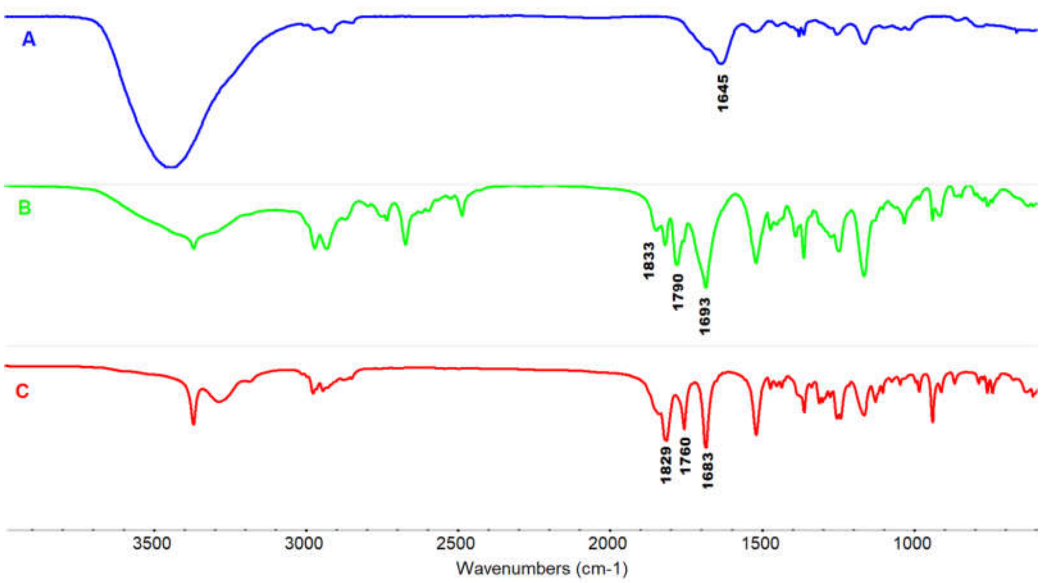

Figure S1. FT-IR spectra: (**A**) of Boc-Lys(Boc)-OH precursor compound; (**B**) after 2.5 hours of reaction; (**C**) of the final product N^{ε}-Boc-L-Lys NCA after recrystallizations.

The pure monomer was then characterized by ^{1}H-NMR spectroscopy in $CDCl_3$ (Figure S2). It is observed that all peaks are attributed to hydrogen atoms of the NCA, while the integrations of the peaks approach the theoretically predicted values. Specifically, ^{1}H-NMR (400 MHz, $CDCl_3$, δ, ppm): 1.46–1.60 (13H, 9H from BOC group –C($\underline{CH_3}$)$_3$, 4H from –HN–CH_2–$\underline{CH_2}$–$\underline{CH_2}$–CH_2–), 1.85–2.04 (2H, from –HN–CH_2–CH_2–CH_2–$\underline{CH_2}$–), 3.11–3.19 (2H from –HN–$\underline{CH_2}$–CH_2–CH_2–CH_2–), 4.32–4.35 (1H, N–$\underline{CH}$–CO of the anhydride ring), 4.65 (1H, CH–$\underline{NH}$–CO of the anhydride ring), 6.62 (1H, –CH_2–$\underline{NH}$–CO– of the ε-amine), FT-IR (thin film): 1829, 1760 cm^{-1} (ν CO, NCA), 1683 cm^{-1} (ν CO, BOC protecting group). By identifying all the hydrogens of the spectrum, the successful synthesis as well as the purity of the final NCA was confirmed.

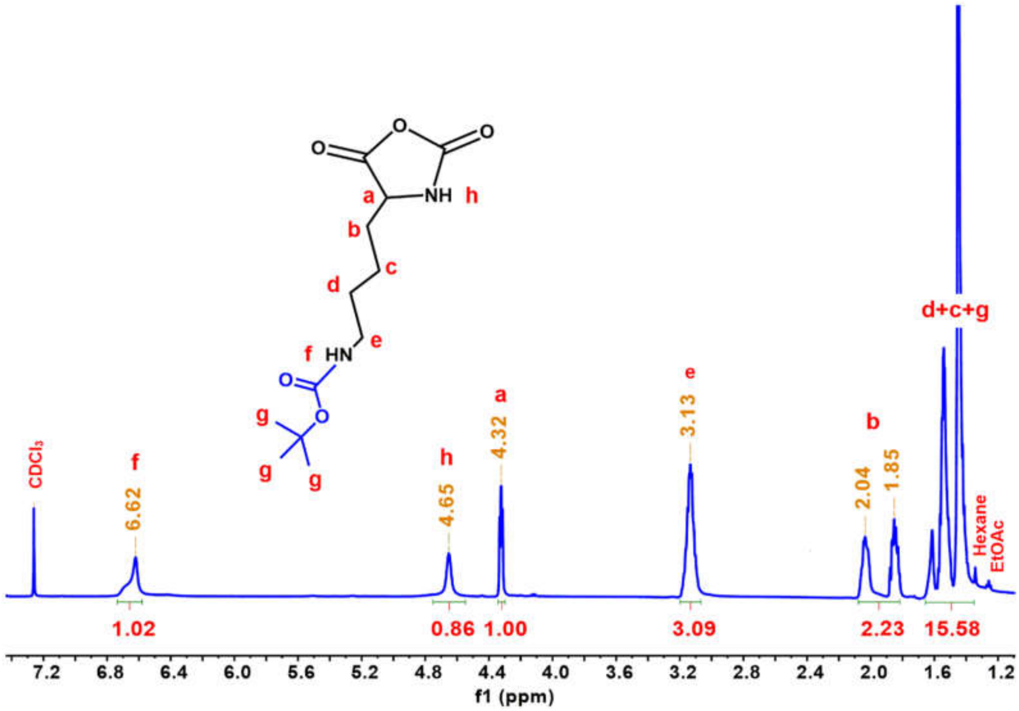

Figure S2. ^{1}H-NMR spectrum of the final *N*ε-Boc-L-Lys NCA in $CDCl_3$.

Characterization of Nim-Trt-His-NCA

Initially, an FT-IR spectrum of the precursor amino acid of histidine (Boc-His(Trt)-OH) was taken (Figure S3A). The characteristic peak at 1710 cm^{-1} is due to the vibration of carbonyl (C=O) of the amino acid of histidine. Then, $SOCl_2$ was used as a chlorination agent to carry out the cyclization reaction and the reaction was left for 2.5 hours. The addition of $SOCl_2$ must be carried out at low temperature, because at high temperatures there is a risk of detachment of trityl-protective groups. The completion of the reaction was confirmed by FT-IR spectroscopy (Figure S3B). A reduction of the peak at 1710 cm^{-1} and the appearance of two peaks at 1790 cm^{-1} and 1857 cm^{-1}, which correspond to the symmetrical and asymmetric vibration of the NCA carbonyls, indicate the successful synthesis of *N*-carboxy anhydride. A characteristic peak of imidazole chloride vibration at 1622 cm^{-1} is also observed. The complete capture of HCl from the imidazole ring of the NCA was achieved by using a stoichiometric amount of triethylamine to form the $Et_3N{\cdot}HCl$ salt, which was immediately removed from the desired product by filtration. In the FT-IR spectrum of the final NCA (Figure S3C) the peak at 1710 cm^{-1} is absent, indicating that the precursor amino acid of histidine has been fully consumed. The lack of the peak at 1622 cm^{-1} indicate that HCl has been quantitatively removed after the addition of triethylamine, while the lack of a peak at 1650 cm^{-1}, confirm the success in NCA synthesis. Throughout the synthetic route, the characteristic peaks of the trityl protective groups at 711 and 759 cm^{-1} corresponding to the vibrations of the bonds –CH=CH– of the benzene rings, remained intact.

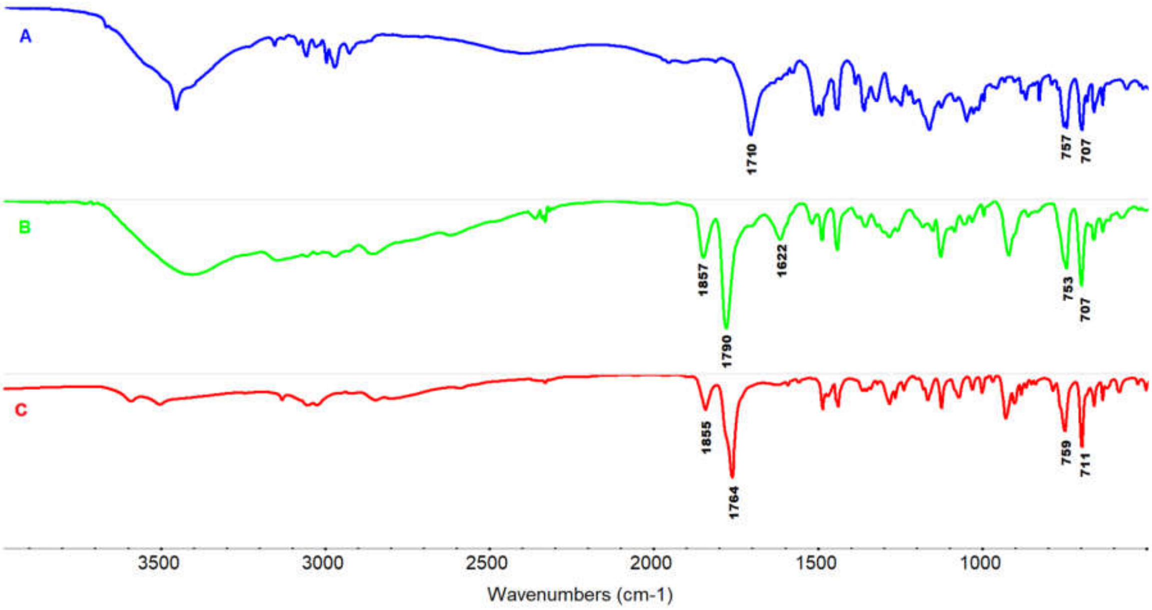

Figure S3. FT-IR spectra: (**A**) of Boc-HIS(Trt)-OH precursor compound; (**B**) after 2.5 hours of reaction with $SOCl_2$; (**C**) final N^{im}-Trt-HIS NCA product after recrystallizations.

In addition, the successful synthesis and high purity of N^{im}-Trt-His NCA were evaluated by ^{1}H-NMR of the received solid in $CDCl_3$ (Figure S4). It is observed that the peaks are all attributed to hydrogen atoms of the NCA, while the integrations are exactly as predicted theoretically. ^{1}H-NMR (400 MHz, $CDCl_3$, δ, ppm): 2.96–3.18 (2H, –<u>CH₂</u>–imidazole–Trt), 4.55–4.57 (1H, –N–<u>CH</u>–CO of the anhydride ring), 6.66 (1H, CH–<u>NH</u>–CO of the anhydride ring), 7.09–7.35 (16H, 15H from trityl group and 1H N–CH=C of imidazole ring), 7.39–7.68 (1H, N–<u>CH</u>=C, 1H, N–<u>CH</u>=N of imidazole ring), FT-IR (thin film): 1855, 1764 cm^{-1} (νCO, NCA), 711 cm^{-1}, 759 cm^{-1} (ν=C–H out of plane bend).

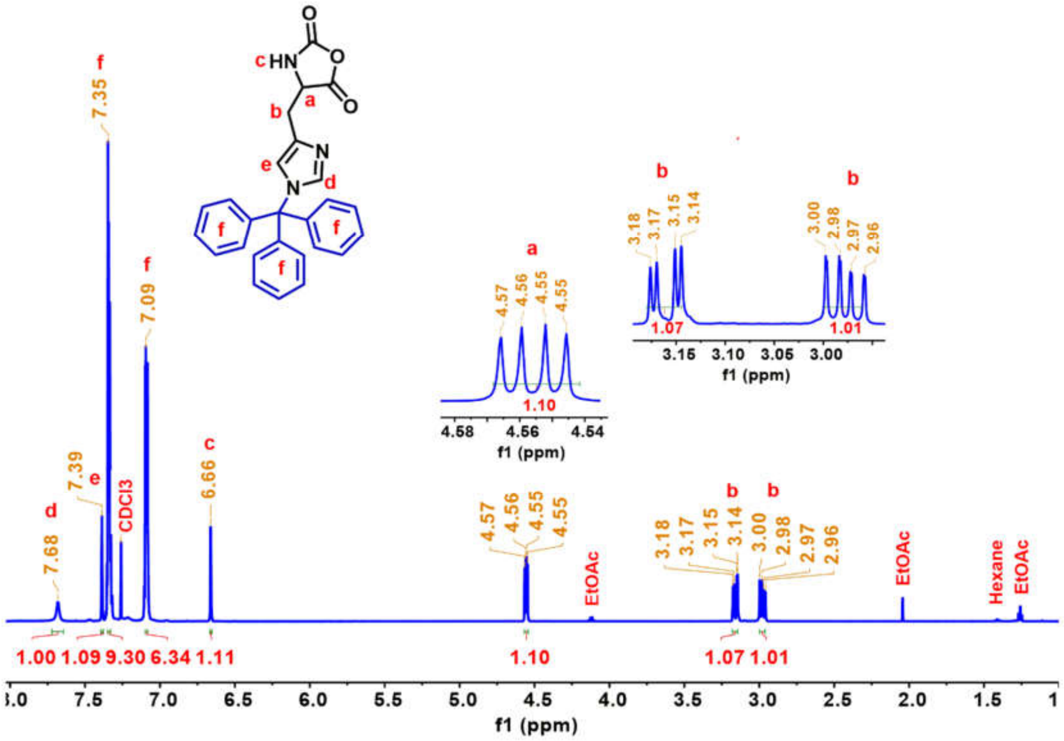

Figure S4. ^{1}H-NMR spectrum of the final N^{im}-Trt-L-His NCA in $CDCl_3$.

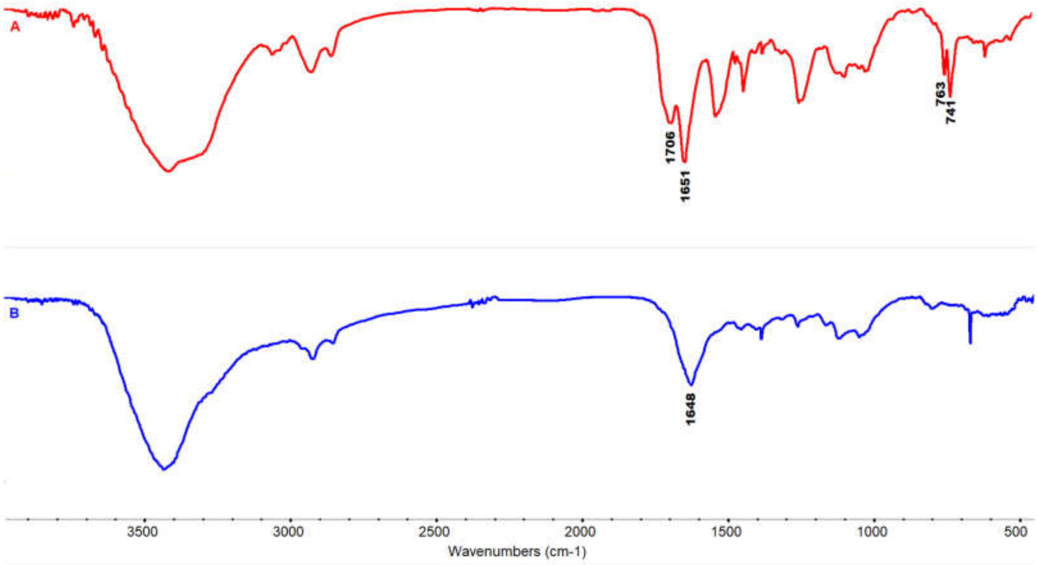

Figure S5. FT-IR spectra of: (**A**) the protected Poly(N^{ε}-Fmoc-L-Lysine)$_{78}$; (B) the deprotected Poly(L-Lysine)$_{78}$ homopolymer.

References

1. Hernández, J.R.; Klok, H.A. Synthesis and ring-opening (co) polymerization of L-lysine N-carboxyanhydrides containing labile side-chain protective groups. *Journal of Polymer Science Part A: Polymer Chemistry* **2003**, *41*, 1167-1187.
2. Hanski, S.; Houbenov, N.; Ruokolainen, J.; Chondronicola, D.; Iatrou, H.; Hadjichristidis, N.; Ikkala, O. Hierarchical Ionic Self-Assembly of Rod−Comb Block Copolypeptide−Surfactant Complexes. *Biomacromolecules* **2006**, *7*, 3379-3384, doi:10.1021/bm0606770.
3. Mavrogiorgis, D.; Bilalis, P.; Karatzas, A.; Skoulas, D.; Fotinogiannopoulou, G.; Iatrou, H. Controlled polymerization of histidine and synthesis of well-defined stimuli responsive polymers. Elucidation of the structure-aggregation relationship of this highly multifunctional material. *Polym. Chem.* **2014,** *5* (21), 6256.
4. Benoiton, N.L. *Chemistry of peptide synthesis*; CRC Press: **2016**.
